# From One Medicine to Two: The Evolving Relationship between Human and Veterinary Medicine in England, 1791–1835

**DOI:** 10.1353/bhm.2017.0058

**Published:** 2017

**Authors:** Abigail Woods

**Keywords:** veterinary medicine, human medicine, one medicine, professionalization, reform, Britain, nineteenth century, comparative anatomy

## Abstract

This article offers a novel perspective on the evolving identities and relationships of human medicine and veterinary medicine in England during the decades that followed the 1791 foundation of the London Veterinary College. Contrary to the impressions conveyed by both medical and veterinary historians, it reveals that veterinary medicine, as initially defined, taught and studied at the college, was not a domain apart from human medicine but rather was continuous with it. It then shows how this social, cultural, and epistemological continuity fractured over the period 1815 to 1835. Under the impetus of a movement for medical reform, veterinarians began to advance an alternative vision of their field as an autonomous, independent domain. They developed their own societies and journals and a uniquely veterinary epistemology that was rooted in the experiences of veterinary practice. In this way, "one medicine" became "two," and the professions began to assume their modern forms and relations.

[Other P-494]

In his popular and influential book *Outlines of the Veterinary Art* (1802), veterinarian Delabere Blaine committed himself to improving a "branch that has sprung from, and must grow with medicine as its parent stock."^1^ Claiming that until recently, the art had advanced mainly "by stealth . . . usually by the exertions of some enlightened physician or surgeon," he aimed to complement the work of the London Veterinary College (LVC) in raising its dignity and utility.^2^ He wrote for the three groups he thought likely to study the art: surgeons—who had already "travelled three fourths of the road towards making a good veterinarian," "persons of fortune with enlarged minds and extended educations," and "farriers . . . or persons intending to profess veterinary medicine."^3^ Preferably they should attend the LVC, but if this was not possible they should study general descriptions of the human body, dissect horses "at the tan yard or kennel," and read medical literature on physiology, pathology, comparative anatomy, chemistry, materia medica, and farriery. After this, the farrier lacked only "experience and practice to perfect him."^4^

Written just a decade after the 1791 creation of the LVC—an event that veterinarians today regard as the foundation of their profession in Britain—Blaine's account draws attention to an important and understudied aspect of medical history: the relationships between human and veterinary domains. Whereas today these exist as separate fields involving different professions, institutions, and human/animal subjects, Blaine's text suggests at the turn of the nineteenth century their relationships were more fluid. Then, the "veterinary art" existed as a "branch" of human medicine, grounded in knowledge of humans and partially populated by surgeons. Blaine's career trajectory reinforces this portrayal. He was originally apprenticed to a surgeon-apothecary, then enrolled as a medical student at the Borough hospitals, London, where he assisted the physiologist, Dr. Haighton, in his animal experiments. He then worked temporarily as a teacher at the LVC, before leaving to take up human civilian then military surgery. Finally he became a veterinary practitioner and author.^5^ He was not unusual in this regard. During the previous century, elite providers [Other P-495] of equine health care had followed similar paths, training initially as surgeons and occasionally physicians.^6^

With the exception of Michael Mackay, whose doctoral thesis provides an illuminating analysis of these elite equine healers and the horse infirmaries they established in response to the growth of human infirmaries,^7^ medical historians have generally failed to recognize the interpenetration of human and animal health care in Britain in the decades around 1800. Despite the well-documented epistemological breadth of late eighteenth-century medicine, its inclusion of pursuits remote from medical practice,^8^ and the many important roles it awarded to animals—as subjects of experiment, comparative anatomy, and natural history and as sources of cowpox lymph for use in human vaccination^9^—histories of medicine remain largely focused on human healers and their human patients,^10^ while animal healers and patients are compartmentalized into the separate sphere of veterinary history.^11^ Authors either fail to consider the possibility that veterinary medicine lay within the boundaries of human medicine, or else reject it outright on the grounds that medical men looked down on and showed little interest in animal health and healing.^12^

The latter opinion is open to challenge because it unproblematically reproduces the rhetorical claims of men like Blaine, who argued that veterinary improvement was needed because the field was degraded and beneath the dignity of a gentleman.^13^ While the early veterinary art did [Other P-496] have disagreeable associations with empirical farriery, surgery, too, was struggling to throw off its image as a manual craft. Improvers of both fields thought the solution lay in the development of scientific principles, and in pursuing them, surgeons did not restrict themselves to the human species.^14^ This is acknowledged by veterinarians writing the history of their profession, who recognize that surgeons played important roles in the early years. However their interpretations of this phenomenon are skewed by the presentist assumption that the veterinary profession was always destined to assume its modern shape and significance. Concluding that participation of medical men was a necessary, temporary stepping stone to veterinary autonomy, they celebrate surgeons like John Hunter who helped to establish the LVC, while denigrating others who refused to withdraw from that institution at the "appropriate" time.^15^

In attempting to address these deficits in historical understanding, this article has three main goals. First, it aims to advance conceptions of what constituted human medicine in England at the turn of the nineteenth century by taking seriously its relationship with the "veterinary art." Second, by analyzing how that art was perceived and shaped by its early promoters and participants, it offers a novel perspective on the history of veterinary medicine. Third, having described the highly integrated nature of human medical and veterinary domains circa 1800, it aims to explain their subsequent separation, largely from the perspective of reforming veterinarians who worked to develop an occupational and epistemological identity distinct from that of human medicine.

As we will see, this separation was under way by 1826, when the third edition of Blaine's *Veterinary Art* proclaimed the "prospect of a new era in medicine, [each field] equally perhaps useful and important to the one as to the other."^16^ It had advanced further by the fourth edition of 1832, in which Blaine wrote the contributions of surgeon-farriers out of history, sidelined the teachings of medical doctors, and referred to veterinarians as a "brotherhood."^17^ In 1844, the award of a Royal Charter set a legal seal [Other P-497] on this transformation by recognizing practitioners of the veterinary art as members of a discrete profession governed by its own Royal College of Veterinary Surgeons, which still performs this function today.^18^ Concurrently, human medicine underwent its own, well-documented transformations as new medical knowledge, cultural values, professional overcrowding, and the wider climate of social, political, and religious reform prompted the questioning and refashioning of medical institutions, identities, and epistemologies.^19^ This article draws heavily upon historical accounts of the latter events in order to make sense of and demonstrate their connection with changes in the veterinary field.

In this analysis, "veterinary" will be treated as an actor's category. It was first defined as an occupation and as a field of enquiry in Britain by men who created, taught and studied at the LVC.^20^ Students who passed its examinations were deemed "qualified to practice the veterinary art" and assumed the title "veterinarian," or (after army commissions were introduced in 1795) "veterinary surgeon." They either joined the army, or entered a competitive market for animal healing that was already populated by farriers (who treated livestock as well as horses), horse doctors, cow leeches, blacksmiths, druggists, cunning folk, country surgeons, and animal owners.^21^ Along with their teachers, these men wrote the first texts on the "veterinary art," a term that was used interchangeably with "veterinary medicine" and less commonly "veterinary surgery." Unlike in human medicine, there was no distinction between the practice of physic and surgery.

While veterinarian-historians have tended to regard veterinary medicine as discontinuous with the preexisting form of animal healing known as farriery,^22^ Mackay has argued convincingly for its overlap with the "improved farriery" pursued by elite eighteenth-century medical men. In the closing decades of the century, these men—who were mostly surgeons—established [Other P-498] equine infirmaries modeled on human hospitals as key sites for the teaching and practice of improved farriery. The LVC was organized along similar lines. It, too, was founded by subscription and focused on the horse. It coexisted and competed with the institutions of improved farriery, and against all the odds, it survived while they did not.^23^ Its promoters may have adopted the term "veterinary" in order to create a (largely artificial) distinction from farriery, and to position it alongside the French Ecoles de Veterinaire in Lyons (est. 1762) and Alfort (est. 1765). Their founder, Claude Bourgelat, had adopted the title for that very reason, and the LVC's first principal, Charles Vial de Saintbel, had trained under him at Lyons.^24^

This article draws on the books, journals and correspondence of the first English men to define themselves as veterinarians in order to determine how they perceived the veterinary art and its relations to human medicine, why they participated in it, and how they contributed to its evolution over time. Scottish veterinary medicine—which grew out of the school founded in Edinburgh in 1823 by farrier William Dick—is beyond the scope of analysis, although this would make for an instructive case comparison.^25^ The focus is more on the image of English veterinary medicine than on its actual practice, which remains a subject for further investigation. However, for the purposes of this article it is important to note that while veterinarians sometimes treated farmed livestock, pets, and occasionally exotic animals, horses were the most common veterinary patient, and in military contexts and the LVC infirmary other species were seldom if ever seen. The most common condition treated by the LVC was lameness, which had multiple causes and was generally managed through surgical interventions and the use of specially designed shoes, fitted by blacksmiths under veterinary supervision. Horse patients were also treated with physic for various internal ailments, and frequently bled.^26^

Through exploring the early participation of medical men in the formation and running of the LVC, the first section of this article will reveal that circa 1790 to 1810, veterinary medicine lay within the broad domain of human medicine. The second section explores how its identity was [Other P-499] affected by the early nineteenth-century campaign for medical reform, which included a campaign to reform the LVC. It concludes that despite major shifts in medical identity, culture, and epistemology, many medical men continued to locate veterinary medicine within their sphere of concern. However, practicing veterinarians were beginning to perceive of it as a domain apart. The remainder of the article explores and explains this perception with reference to veterinary social organization, occupational identity, and the emergence of a new, specifically veterinary epistemology within the fields of farriery and comparative anatomy. It will reveal that paradoxically, in their aspirations to separate from medicine, and in the strategies they devised to achieve this goal, veterinary reformers took their lead from medical reformers. Therefore the eventual establishment of veterinary medicine as a domain distinct from medicine resulted, in part, from the very influence that medicine exerted upon it.

## One Medicine

In 1792, the LVC opened its doors to pupils. Established with the objectives of teaching the veterinary art, establishing a veterinary infirmary, and encouraging the pursuit of veterinary science, it represented the culmination of a drive to improve farriery initiated several years earlier by the Odiham Agricultural Society. This small, provincial organization had pursued this goal as part of its wider agenda for agricultural improvement, which was a popular Enlightenment ambition. The decision to found a college derived from a chance meeting between one of its members, the wealthy Quaker, scholar, and reformer Granville Penn, and the French veterinarian Charles Vial de Saintbel, who was already planning such a college. Saintbel had moved to England in 1788 following his studies at the Ecole de Veterinaire in Lyons and a post as comparative anatomy demonstrator at the Montpellier medical school. In 1789 he made a name for himself in horse-racing circles after dissecting the famous British racehorse Eclipse.^27^ He went on to exhibit Eclipse's skeleton in his home. He also announced his intention to lecture gentlemen on horse anatomy, physiology, and disease, and to accept as lodgers those "inclining to make extraordinary improvements in the veterinarian art."^28^ Penn helped to set these plans on a more formal setting. A London-based committee was created and raised funds by subscription for a veterinary college. It appointed a president (the Duke of Northumberland), vice presidents, [Other P-500] directors, and board members, and took a house in Camden Town where Sainbel was installed as principal.^29^

Medical men were enthusiastic participants in the creation of the LVC. Its vice presidents included the prominent surgeon John Hunter (who died in 1794), Sir George Baker (physician to the king), and Sir William Fordyce (a prominent London physician), as well as three earls, a lord, and a baronet. The board contained nobles, gentlemen, and a number of other medical men.^30^ It formed a medical experimental committee to suggest experiments to be carried out at the LVC. Populated wholly by medical men, particularly surgeons, the committee's remit was soon extended to staff appointments, student examinations, and awarding signed diplomas to successful candidates.^31^ Many of its members were associates and former pupils of John Hunter, and would dominate the London hospitals and the Royal Colleges of Surgeons and Physicians for decades to come. Their reasons for participating in the LVC are best understood by reference to what historian Michael Brown has termed the culture of "medico-gentility." This offered a way for medical men—and particularly surgeons, who were trying to shed their reputation as uncultivated manual workers—to differentiate themselves at a time when medicine had not yet developed into a bounded vocation characterized by compulsory licensing, specialist education, the exclusive possession of scientific expertise, and a strong professional identity. Orienting themselves toward polite society rather than the medical collective, medical gentleman sought gentility through active participation in civic life, the cultivation of social networks, and investment in various enlightened pursuits that formed part of—but were not exclusive to—the broad epistemological domain of medicine.^32^

These pursuits included a number of animal-related areas of enquiry that connected to veterinary medicine. As already noted, elite equine farriery had begun to attract converts from medicine in the early eighteenth century. Amid growing interest in large-scale horse racing, selective horse breeding, hunting on horseback, and the performance of cavalry horses—which generated a market demand for elite horse healers and fueled aristocratic interest in the LVC—physicians like Henry Bracken and surgeons such as William Gibson and William Osmer worked to [Other P-501] identify its principles and to refashion it from an empirical practice into a polite gentlemanly art. Inspired by the growth of hospitals and their use in medical education, other eighteenth-century surgeons wrote manuals, founded horse infirmaries, and offered lectures on the structure, function, and diseases of the horse.^33^

Agricultural improvement was another area of medical interest. Working alongside the landed gentlemen whose estates were being transformed through enclosure, certain eighteenth-century medical men sought to uncover and communicate the principles of improvement through agricultural and horticultural societies, texts, and pamphlets, and the conduct of trials and experiments.^34^ Livestock were important to this agenda, but their improvement was threatened by repeated epidemics of the highly contagious and fatal disease known as cattle plague or rinderpest. Medical men made considerable efforts to elucidate, prevent, and control this disease. Its ravages gave impetus to the LVC by inspiring the Odiham Society—whose members included several medical men—to pursue the improvement of farriery.^35^

There was also a long-standing medical tradition of studying animal bodies through observation, dissection, collection, and experiment. These activities overlapped with the polite gentlemanly art of natural history. Medical men, particularly surgeons, worked to identify the similarities and differences between humans and animals; to understand how bodies functioned in health and disease; to test out surgical and therapeutic interventions; to construct hierarchical classificatory systems that demonstrated human-animal relationships; and to convey this information to students and interested onlookers.^36^ The surgeon John Hunter, whose work was widely credited with raising the scientific status of surgery, was a particular enthusiast. He inspired pupils and colleagues to follow suit, and join him in supporting the LVC. His enormous collection of human and animal specimens attracted the attention of nobles and gentlemen. [Other P-502] Some built collections of their own or conducted animal experiments, and as "men of science," they were eligible for election to the Royal Society.^37^ Some of its members pledged support for the LVC.^38^

Medical and gentlemanly participants in the above activities valued them not only for their contributions to knowledge and practice, but also because of their moral worth in enhancing understandings of God's plan, nurturing improvement, advancing science, ordering society, and preventing animal suffering. The foundation of the LVC provided an opportunity for them to integrate and advance these various agendas, and thereby elaborate their identities as enlightened, benevolent gentlemen. Its medical supporters were not particularly concerned with practicing veterinary medicine themselves. Rather, as in their concurrent efforts to improve human surgery, they aimed to establish and propagate a set of enlightened principles to guide its practice by others, and through which they could establish their own gentlemanly status.^39^ Their participation also enabled them to forge social connections with elite supporters (who were potentially lucrative patients), and to display the relevance of their expertise to the LVC's advertised objective: "to amend, and bring into a regular system, that important branch of medicine which regards the treatment of diseases incident to horses and other cattle, and which has hitherto been neglected, and much abused in this country."^40^

Surgeons also exerted considerable influence over the staffing and running of the LVC. Although Saintbel, the first principal, was not a surgeon, he was in post for only two years. In 1793 he died suddenly, probably from the horse disease glanders.^41^ By then, he had fallen out with and caused the departure of Blaine, who had earlier accepted an invitation to teach at the LVC because he was "enthusiastically attached to animals . . . as well as to natural history and comparative anatomy."^42^ Following a lengthy search, the medical committee selected two new principals, both surgeons. William Moorcroft had trained also as a veterinary surgeon at Lyons on the advice of John Hunter, and ran a veterinary practice in Oxford Street. After just six weeks in post he resigned citing ill health, leaving the LVC in the sole charge of Edward Coleman until his death in 1839.^43^[Other P-503]

Coleman had studied under Henry Cline, surgeon to St Thomas's Hospital and a member of the LVC's medical committee. With fellow pupil Astley Cooper (a future member of the LVC's experimental committee), he had attended John Hunter's lectures and performed animal experiments, most notably to investigate the suspicion that death by hanging and drowning was caused solely by mechanical blockage of the windpipe. He had also enquired into the comparative anatomy of the eye.^44^ The medical committee declared him to possess "great knowledge in the veterinary art."^45^ He had no practical experience of it—indeed Astley Cooper claimed he would "burn his fingers if he tried to burn on a horseshoe"—but his talents "were of a higher and more refined kind."^46^ As a qualified surgeon and "man of science," he grasped the learned principles of the art, and was therefore considered able to teach and improve it.^47^

The education offered by Coleman focused almost exclusively on the horse. It resembled and overlapped with that of medical students, who were increasingly supplementing their traditional apprenticeships with a period spent walking the hospital wards and attending lectures offered by hospital doctors and other entrepreneurial medical men.^48^ Most veterinary students also undertook apprenticeships. They came to London to add knowledge of principles to their experience of practice. They paid Coleman twenty guineas to attend his lectures on the veterinary art, training in (horse) dissection, and clinical teaching in the LVC infirmary on horse patients belonging to wealthy college subscribers. Coleman also encouraged pupils to attend external lectures for medical students on human anatomy, comparative anatomy, physiology and surgery, materia medica, chemistry, and the practice of physic. These were provided by members of the LVC's medical committee and their associates, who had followed John Hunter's lead in inviting veterinary students to attend free of charge in the wake of Saintbel's death. This provision reduced the need for lecturing at the LVC.^49^ Members of its medical committee were also responsible for examining veterinary students and signing the diplomas of those who passed. Their national reputation was intended [Other P-504] to secure public confidence in diploma holders and elevate the status of the veterinary art.^50^ It also reinforced the idea of veterinary medicine as a branch of human medicine.

This idea was consolidated through the enrolment of surgeons and surgical trainees as LVC students. Governors actively encouraged this, in the belief that their participation would elevate the veterinary art.^51^ Students reluctant to take the whole course could attend fourteen lectures on the veterinary art delivered by Coleman at Guys Hospital from 1801, probably with the support of his friend Astley Cooper.^52^ Various reasons were put forward for why such men should study the veterinary art: their surgical knowledge was highly relevant to it; they would learn how to advance the "study of disease by analogy," to care for their own horses, to treat other people's where no skilled farrier was available, and to enter veterinary practice.^53^ Another compelling reason arose in the context of the French revolutionary wars. In 1795, recognizing the military significance of healthy horses, and in desperate need of money due to Saintbel's mismanagement of LVC finances, governors petitioned Parliament for funds. In return, they promised that Coleman, "a man of science . . . and liberal education," would train one pupil from each regiment in the veterinary art.^54^ Parliament approved the grant. Made annually until 1813, it netted the college over twenty-five thousand pounds in total and reinforced the curricular focus on the horse. In addition the Board of General Officers appointed Coleman chief veterinary surgeon to the cavalry and the Board of Ordnance, and announced its intention to commission veterinarians as officers to each regiment.^55^

Veterinarians thereby gained a new military role, ranked higher than that of regimental farrier and on par with the human surgeon. It was in this context that the term "veterinary surgeon" was adopted.^56^ The [Other P-505] perceived proximity of medical and veterinary domains is illustrated by the board's efforts to attract men "well educated in surgery" into veterinary posts by offering them marginally better pay and conditions. To accelerate their veterinary training, Coleman cut the required duration of attendance at his lectures and infirmary practice to a minimum of three months (with attendance at medical lectures taking place before or afterward).^57^ Evidence suggests that in the first ten years of the LVC's existence, the majority of the 101 students who qualified were surgeons. Nearly half of the total entered the army, thereby achieving status and a respectable wage.^58^ This situation provided Coleman with an opportunity to publicly display his enlightened credentials. He announced that the commissioning of veterinary officers had raised the veterinary art "from contempt to respectability" by inducing "medical students of liberal education, to devote their services to its improvement."^59^

A close reading of veterinary texts written circa 1798 to 1810 reveals that early veterinarians—many of them surgeons who took up army commissions—adhered to the same cultural values and epistemological outlooks as the LVC's medically trained promoters and teachers. These authors situated veterinary medicine firmly within the domain of human medicine, and sought, through its improvement, to advance their own identities as gentlemanly members of polite society.^60^ They highlighted the analogies between human and equine bodies, and human and veterinary medicine, while pointing out the greater difficulty of veterinary medicine on account of the dumb, irrational nature of the horse. Locating themselves within a genealogy of eighteenth-century medical improvers of farriery, whose achievements they documented and evaluated, they presented human [Other P-506] medicine as the arena from which veterinary improvements had and would continue to arise (although this claim was much disputed by practical farriers, who claimed that surgeons' interventions were frequently erroneous and impractical).^61^ One author went so far as to claim that "the art never made any progress until it attracted the attention of those who had made special study of human economy. . . . Almost the only rational improvements . . . were either suggested or carried into effect by medical men; and nothing will contribute so much to its perfection as the interest which the profession has lately shewed to it."^62^

Authors opened their volumes with florid dedications to royalty, aristocracy, members of the LVC committee, and Professor Coleman, and directed them at audiences of gentlemen, the nobility, medical men, veterinarians, and farriers. They included lengthy, erudite chapters on history, comparative anatomy, and the principles of horse management in health and disease. Their tone was moralizing and improving. They spoke of the horse as a noble and useful animal, of first importance to the nation and next to the human in dignity. Left in the hands of empirical blacksmiths, grooms, and stable boys, who were ignorant, cruel, prejudiced, and corrupt, its health had been sadly neglected. Such men had disparaged learning, had manipulated their employers, and, for reasons of personal gain, had discouraged the sending of horses to the LVC.^63^ Authors pointed the way to a more enlightened approach, in which owner, horse, and nation would benefit from "the full establishment of rational practice, in which humanity and tenderness are blended with judgement, directed by experience."^64^ In this way, they presented themselves to their publics as learned, benevolent, improving gentlemen.

## The Campaign for Reform

Subsequent decades saw dramatic shifts in the epistemology of human medicine and the culture that had encouraged aspiring medical men to situate veterinary medicine within it. This was part of a wider shift in knowledge and social organization brought about by the disaggregation of broad eighteenth-century modes of thinking and operating into more [Other P-507] specialized, vocationally specific domains.^65^ Historians have explored this shift within human medicine. They reveal how a group of middle-class general practitioners—in which religious dissenters were well represented—turned away from civic society to develop a new collective identity as scientific experts who served the public through the development of useful knowledge. Their challenge to the older culture of medico-gentility and its polite forms of knowledge was lengthy and contentious. Fueled by economic competition between a rapidly expanding body of general practitioners and the burgeoning ranks of druggists and "irregular" healers, it underpinned what historians have labeled "the age of medical reform."^66^

Efforts to refashion medical knowledge and culture were constitutive with a wider reformist agenda, manifesting particularly in the 1820s and 1830s, in bids to outlaw slavery, remove trade monopolies, and bring about electoral reform, the democratization of town councils, and the dismantling of legal restrictions on nonconformists.^67^ Through the medium of journals like the *Lancet* (est. 1823)^68^ and vocationally specific groupings such as the Provincial Medical and Surgical Association (PMSA, est. 1832, which became the British Medical Association in 1856), general practitioners sought to reform medical institutions, structures, and values. As in the wider political arena, they condemned as corrupt the traditional ways of working that relied on patronage and interpersonal connections, and charged their upholders with financial self-interest and the failure to advance society. They called for more democratic, meritocratic, technocratic forms of governance that would promote and reward the development of useful scientific knowledge, and erect barriers between regular and irregular healers. Their particular targets were the leaders of the Royal Colleges of Physicians and Surgeons, and the staff of the London hospitals, whom they charged with nepotism, self-interest, and the failure to value, pursue, and disseminate useful scientific knowledge.^69^[Other P-508]

Since veterinary medicine was widely perceived to be part of human medicine, it was not immune to these developments. In fact it experienced its own campaign for reform, directed against the LVC. By the 1820s, the governors of this institution were largely inactive. Meeting in small numbers once a year, they seemed content to leave the college in the hands of Coleman and the medical examining committee. Members of that committee composed the same elite circle of doctors who were targeted by the campaign for medical reform.^70^ Consequently, the LVC was subjected to criticisms that were almost identical in language and content to those that reformers directed at the London hospitals and the Royal Colleges of Physicians and Surgeons, and via one of the same vehicles: Thomas Wakley's radical weekly, the *Lancet*. Its attack commenced in 1826, when Wakley opened correspondence columns to (largely anonymous) complaints about "abuses" at the Veterinary College, and lent his editorial support to them.^71^

Practicing veterinarians, many of them religious dissenters, were highly active in the campaign for LVC reform. They met in London taverns to voice their complaints and develop plans of action.^72^ They followed Wakley's example, and founded two of their own periodicals in 1828. The short-lived *Farrier and Naturalist* (1828–31) was created by Bracy Clark, a Quaker, surgeon, member of the Linnean Society, and one of the first students to enter the LVC. His language and sentiments directly replicated those of Wakley. The *Veterinarian* was a more moderate publication founded by William Youatt, a former Unitarian minister who had attended the LVC (without taking its diploma) and worked with Blaine for twelve years before taking over his practice.^73^ Both journals published editorial commentaries, correspondence, and scientific and clinical material, whose content reflected their editors' particular interests in the scientific shoeing of horses (Clark), and comparative anatomy and pathology (Youatt).[Other P-509]

The sentiments expressed by LVC reformers (which generated no public response from Coleman and the medical examining committee) indicate their departure from the earlier culture of medico-gentility, and adoption of a new utilitarian and technocratic outlook that privileged scientific knowledge as a route to social progress. Rejecting the earlier notion that medical participation in veterinary medicine would automatically lead to its improvement, they demanded concrete evidence of medical contributions and found it lacking. Not only had the LVC failed to look beyond the horse, but even here it had failed to advance knowledge and practice. According to a *Lancet* editorial, "For the prosecution of physiological enquiry, for the cultivation of comparative anatomy, and for an acquaintance with the diseases of domestic animals . . . no institutions are so well adapted as those termed veterinary colleges."^74^ However, this potential had not been realized, for the LVC had published no lectures, case reports, or scientific findings. "Where are the fruits of 30 years in comparative anatomy and medicine?" asked Bracy Clark's *Farrier and Naturalist*. "Not a single fact has been added by this pompously announced [medical examining] committee to the common stock of zoological knowledge."^75^ Its failure meant that as in human medicine,^76^ veterinary medicine in Britain lagged far behind the continent.

Critics attributed this state of affairs to the LVC's nepotistic culture that rewarded privilege rather than merit. They described Coleman and the medical examiners as a "self-selected" "tyrannical few"^77^ ruling over "one of the most rotten public establishments in England,"^78^ which operated more as "a private school, than as a free and public College."^79^ The substantial public subsidy that Parliament awarded the school had been turned into private profit. Medical examiners pocketed fees from students who they were incapable of examining properly on account of their lack of practical veterinary knowledge, while Coleman's avarice in pursuing private practice as well as his army posts detracted from the delivery of veterinary education.^80^ The LVC's low standards had "deluged the country with pretenders" who knew nothing of the veterinary art and "are laughed [Other P-510] at by blacksmiths and grooms."^81^ The *Lancet* concluded, "With its patronage, funds and means it possesses of promoting the science of zootomy it is disgusting to reflect that it has served only as a mass of corruption to fatten some of the idlest and most unworthy drones."^82^

While the overlap between veterinary and medical campaigns for reform can be explained by reference to their shared targets, the London medical elites, evidence suggests that this was not the only reason. Veterinary reform attracted the attention of medical reformers because they—like the very men they were criticizing—still perceived it to be part of the wider medical domain. Although the earlier diversity of medical knowledge was giving way to more specific forms of vocational expertise, animals and their health and healing still held considerable interest to medical men, but for different reasons than in earlier years. This interest was no longer a marker of polite gentlemanly identity, but a means of advancing knowledge and practice, and (as revealed by Desmond's exemplary account of comparative anatomy)^83^ of mobilizing particular social, political, and religious agendas.

On the grounds that humans and animals were bound by the same fundamental biological laws,^84^ medical men sought to elucidate diseases like rabies and glanders that seemed to transmit between them;^85^ to elaborate species similarities and differences through comparative anatomy;^86^ to advance physiological understandings through animal experiment;^87^ and to treat sick horses belonging to themselves, their patients, and other people.^88^ Their interests were reflected in Wakley's *Lancet*, which regularly published lectures, meeting reports, articles, book reviews, and correspondence on matters concerning animal diseases and veterinary medicine. This continued even after the creation of dedicated veterinary journals. [Other P-511] Men with medical backgrounds also continued to enroll as LVC students, though now in smaller numbers. Coleman claimed to have taught 130 of them by 1830.^89^

By contrast, veterinary reformers, including those who had trained also as surgeons, were beginning to develop a quite different vision of their domain. Although they welcomed the *Lancet*'s criticisms of the LVC, and shared the values of medical reformers (many of whom were also middle-class practitioners and religious dissenters), they did not see themselves as participants in a broader effort to reform medicine. Rather, their ambition was to establish veterinary medicine as an independent sphere, a "sister" profession rather than a child of the medical parent. As the remainder of this article will reveal, this aspiration reflected their growing perception of themselves as a distinctive body of men holding a specific body of expertise, which could be taught, examined, and advanced only by practicing veterinarians.

## Two Medicines

With the end of the Napoleonic Wars in 1815, veterinary opportunities for army commissions diminished. Consequently, more veterinarians sought work in private practice, where they had no monopoly on posts, or position within a predetermined hierarchy. They entered a competitive marketplace populated by many different healers, and in which horses were not the only patients.^90^ To animal owners, the veterinarian's superiority was not self-evident, either socially—because the ranks of LVC students were swelled by the sons of tradesmen and farriers—or practically—because its training focused mainly on principles pertaining to the horse, and could last as little as three months.^91^ This situation led practicing veterinarians to develop a somewhat paradoxical relationship with the LVC. As shown above, they became highly critical of its failure to effect improvements in the veterinary art. At the same time, they identified increasingly with it, as the source of a collective identity that distinguished them from other animal healers.

This identification can be seen in the way that veterinary reformers rewrote history during the 1820s and 1830s to minimize the contributions [Other P-512] of eighteenth-century surgeon-farriers, glorify the creation of the LVC, and attribute all significant advances to it.^92^ Whereas in 1802 Blaine (who did not hold an LVC diploma) claimed that anyone prepared to study the veterinary art could call themselves a veterinarian,^93^ holders of the LVC's diploma subsequently became more jealous of their title.^94^ They even queried Blaine's status, leading him to complain in 1831 of his exclusion from "the brotherhood," and to present his entire biography in an attempt to establish himself as a "legitimate" veterinarian.^95^ Likewise, Youatt felt it necessary to remark in 1828 that without a diploma he might be called "a bastard of the profession" but was nevertheless anxious for its improvement.^96^

The creation of veterinary medical societies encouraged veterinarians to identify more with each other and their alma mater. The first was established in London in 1812 by Thomas Mayer, an LVC student who had already completed apprenticeships with a farrier (his father) and a surgeon. As a supplement to Coleman's teaching, he chose to attend lectures at the Windmill Street School, which led to his membership of the Westminster Medical Society associated with it. Having witnessed its benefits to medical students, he was inspired to create a similar society for veterinarians, which was subsequently brought under the LVC.^97^ Like its many medical counterparts—which were equally important to the development of a collective identity^98^—it met weekly and provided a key forum in which students, practitioners, and teachers presented papers, exchanged views, and forged a shared occupational bond.

In 1828, Youatt and the practitioner William Goodwin founded a second London Veterinary Society. Its rules recognized "all persons engaged in the study or practice of Veterinary medicine" as eligible to become ordinary members. Medical men, including medical lecturers to veterinary students and "physicians or surgeons of eminence, who have [Other P-513] distinguished themselves for their researches in comparative anatomy," could only become honorary members.^99^ Bracy Clark believed they should have been excluded altogether.^100^ A third society formed in 1836.^101^ Meanwhile, as already noted, Youatt and Clark founded their veterinary journals. Modeled upon the *Lancet*, the *Veterinarian* and the *Farrier and Naturalist* published scientific articles, editorial comment, and correspondence that served to educate readers, sharpen their political awareness, and build a sense of community. Blaine saw their creation as the most important event in veterinary medicine since the foundation of the LVC.^102^

At the same time, veterinary student attendance at human medical lectures diminished. Having earlier trumpeted their value, by 1831 Blaine was claiming that they were merely "a secondary consideration" that students should consider only after completing their veterinary lectures.^103^ Other, more relevant forms of education had emerged. From 1829, Youatt—who emerged as the most vocal and long-standing critic of Coleman's almost exclusive focus on the horse—offered a course of lectures on the anatomy, physiology, and diseases of domestic animals with the assistance of William Percivall. These were provided at the invitation of London University, a new, nondenominational, utilitarian institution directed toward professional improvement. Offered to both veterinary and medical students, the lectures were acclaimed by and published in the *Lancet*.^104^ Shortly afterward, Charles Spooner (Youatt's former assistant) and William Morton (the LVC's dispenser) followed the example of many medical lecturers and established private classes in veterinary anatomy and pharmacy outside the walls of the LVC.^105^ Youatt also recommended veterinary students to attend the London University course on comparative anatomy given by Robert Grant, who was appointed in 1828 to England's first full-time chair in the subject. A number of other medical men began to offer comparative anatomy courses at around the same time.^106^ [Other P-514] These changes in veterinary sociability and education were both responses to and vehicles for a new veterinary epistemology. They grew out of experiences in practice, and helped to fashion such experiences into the bedrock of veterinary identity. As historians have shown for other forms of scientific knowledge in this period,^107^ the new veterinary epistemology was inherently political. It was a utilitarian body of knowledge that advertised the merits of practice over theory, and actual experience of animals over analogical reasoning about them. Developed by veterinary reformers, it aimed to set distance between what they considered to be veterinary medicine, and the domain as envisaged by both older medical elites and medical reformers. It was most evident within equine farriery and comparative anatomy. This was no coincidence because, as shown above, medical interest in both fields predated the LVC's creation and continued to develop alongside it, lending support to the idea of veterinary medicine as a branch of human medicine. By creating their own versions of them, veterinary reformers sought to challenge and overturn this perception.

Fundamental to farriery was the correct mode of shoeing a horse, which was performed to protect horses' feet and to prevent or manage lameness, one of the key health problems of the day. Eighteenth-century efforts to improve farriery had involved the development of new principles of shoeing and designs for horse shoes.^108^ Coleman followed in this tradition. He disseminated his views on shoeing and the horse's feet via student lectures,^109^ and in his only publications, which appeared soon after his appointment to the LVC.^110^ As army veterinary surgeon, he oversaw the application of the shoes he designed to the cavalry, and encouraged their adoption by the general public through establishing forges around London that were run by former students.^111^ Farriers were quick to ridicule [Other P-515] his methods.^112^ Veterinarians appeared to accept them initially, but as the culture of medico-gentility fractured and reforming sentiment took hold, they subjected them to public scrutiny and criticism, while proposing rival principles and shoe designs.^113^

During the 1810s and 1820s, fierce debates on the relative merits of these systems played out within the medical, veterinary, sporting, and popular press.^114^ It proved impossible to reach consensus on the correct way to shoe a horse. This was partly because some of the key protagonists (Powis, Goodwin, and Blaine) were business rivals, running practices and forges within a small part of London's West End, where they competed with each other and with farriers. It was also because views on shoeing were informed by political and religious sentiments. In the age of reform, opinions on the ideal relationship between hoof and shoe reflected protagonists' diverse beliefs about the ideal relationship between state and citizens, and between God and his subjects.

As a conservative member of the establishment, Coleman had looked to tradition for inspiration on shoeing. Taking up the shoe invented by La Fosse, a farrier to the French king, he made adjustments to ensure that it would not oppress the hoof's natural functions—in the manner that absolute rule had oppressed the French populace—but rather support and protect them, just as British citizens were supported and protected by the rule of constitutional law. The emphasis he placed on the need to prepare the hoof to accept the shoe resonated politically with resistance to the concept of popular sovereignty.^115^ By contrast, the Quaker Bracy Clark, who ran a long-established practice in London's East End, claimed that shoeing was fundamentally problematic because it used nails that constrained the natural movement of the foot and caused injury. In reflection of his radical liberal politics, he advocated that shoes be abandoned, or [Other P-516] replaced with his own invention that allowed the foot to move freely and naturally.^116^ Blaine, also a dissenter, represented the pragmatic, utilitarian middle ground. He believed that while shoeing was necessary to protect the hoof, no single shoe fitted all horses, therefore a selection should be made according to the type and use of horse, with as "little departure from nature as circumstances can justify."^117^ Other veterinarians arranged themselves along this political and religious spectrum.

Although they failed to agree with each other, veterinary reformers united in opposition to Coleman. They claimed that his methods had harmed horses and profession alike, and were pursued purely for reasons of personal financial gain. They believed his approach to shoeing to be entirely flawed because he had no practical skill in shoeing horses. Instead, he worked on the basis of "false theory," constructed on the basis of anatomical analogies with the human body. By contrast, their methods had been developed through long experience and experiment with horses within military settings and veterinary practice, and were designed solely for the benefit of the public. For Clark, Coleman's refusal to acknowledge his failings revealed all that was wrong with "protected incorporations of men, who have interests at variance with the science they profess, and who view invidiously any knowledge or discovery not originating with themselves."^118^

Meanwhile, in the *Veterinarian* and in lectures to London University, Youatt and Percivall advanced a distinctively veterinary concept of comparative anatomy, which deviated from that held by medical elites and medical reformers. As Desmond has shown, comparative anatomy was a key battle ground in the campaign for medical reform. In correlating structure with function, medical elites drew on Cuvier and Paley's natural theology to argue that nature—and by extension, society—was perfectly designed and sanctioned by God. By contrast, in perceiving animals to be formed on the same basic plan, but to vary in how they developed from it, reformers drew on the ideas of Lamarck, Geoffroy Saint-Hilaire, and Romantic naturphilosophie to propose that nature and society were progressive and changing.^119^

In some ways, Youatt's views were closer to those of medical elites than those of medical reformers. He preached upon Paley in 1803,^120^ incorporated [Other P-517] his perspectives into his London University lectures,^121^ and utilized Cuverian classification in his books on the dog, horse, and cow.^122^ However, he differed from both groups in one crucial respect. Whether they adhered to the radical notion of self-organizing matter, or to the Paleyite belief in God as the divine architect, medical comparative anatomists were primarily concerned with discovering the universal principles common to all living things. Their search for these principles—which formed part of their wider efforts to raise the status of surgery by making it scientific—often began with very simple animals, on the basis that human complexity tended to obscure general laws, and that there was sufficient analogy between human bodies and those of lower animals to permit inference from one to the other.^123^

By contrast, as their views on both farriery and comparative anatomy reveal, veterinary reformers were extremely hostile to analogical reasoning. According to Youatt, "reasoning from analogy is here dangerous and inadmissible. We appeal to facts, and to facts alone we bow."^124^ These facts derived largely from domesticated animals, and in collecting them, veterinarians aimed to uncover the differences between species rather than the similarities sought by medical men. This search for difference had obvious political connotations. In subsuming the differences between species, medical men had subsumed veterinary within human medicine. Through elevating species difference, veterinary reformers sought to create a distinctive and independent identity for their field.

There were several ways in which a comparative anatomy focused on species difference advanced the veterinary political agenda. First, it lent a scientific character to the veterinary art and its practitioners, which helped to set it apart from empirical farriery and on a par with human medicine.^125^ Second, because what is "different in a state of health will be different in a state of disease,"^126^ comparative anatomy had practical utility. For Youatt, veterinary medicine was "comparative anatomy made to bear [Other P-518] upon pathology. It is comparative anatomy brought home to practice."^127^ It followed that "to practice the veterinary art in all its branches, the veterinary surgeon must be a comparative anatomist."^128^ In support of these points, veterinarians frequently cited the comparative anatomy lecture delivered to the Royal College of Surgeons in 1818 by William Lawrence,^129^ a surgeon, reformer, and cofounder of the *Lancet*: "Comparative anatomy bears the same relation to the veterinary art that human anatomy and physiology do to medicine. . . . The peculiarities in organic structure and functions of particular genera or species lead to corresponding peculiarities in their disorders or derangements. Hence, a rational treatment of the disorders incidental to animals, presupposes a knowledge of the generic and specific characters of internal organization."^130^

Third, practicing veterinarians saw themselves as uniquely capable of advancing this form of comparative anatomy and the associated domain of comparative pathology through their experiences of sick animals. These experiences were extremely extensive: in 1833 alone, Youatt's practice recorded 1,748 cases, including 426 horses, 1,066 dogs, and 144 zoo animals.^131^ They enabled veterinarians to uncover "the most unexpected and inexplicable difference in the diseases to which the same organs were exposed, and the effect of certain medicines."^132^ Through publishing select reports on his cases in the *Veterinarian* under the heading "comparative pathology,"^133^ Youatt sought to illustrate "the different character of disease in animals of different structure, food and habits."^134^ He expressed hopes that such contributions to "knowledge of comparative pathology, that inexhaustible mine of medical improvement" would vindicate veterinary claims to be "distinctly separate yet closely allied" to medicine.^135^

Finally, comparative anatomy provided veterinary reformers with a weapon to attack medical men who claimed expertise in what they considered to be their domain. They attempted to undermine Coleman's [Other P-519] position at the head of the LVC by highlighting how, in focusing his teaching almost exclusively on the horse, he had failed to appreciate the importance of comparative anatomy, to advance its development, and to provide students with a proper grounding in it.^136^ They also argued that while medical examiners claimed expertise in comparative anatomy, their failure to understand the particular differences between species made them incapable of pronouncing on veterinary students' competence to practice. Without direct experience of sick animals, these examiners, like the medical men who dabbled in veterinary practice, could only draw deductions through analogical reasoning, which was dangerously misleading. It was therefore essential that they cede such roles to practicing veterinarians.^137^ In this way, veterinarians constructed an epistemological basis for their political campaign to win independence from human medicine.

## Conclusion

In exploring the evolving relations between human medicine and early veterinary medicine in Britain, this article offers a significantly new interpretation of both of their histories. In the first place, it shows that veterinary medicine, as initially defined, taught and studied at Britain's first veterinary school, was not a domain apart from human medicine, but rather was continuous with it. This continuity was social, cultural, educational, and epistemological. Medical men, including some of London's most prominent surgeons, molded the early development of veterinary medicine through assuming roles as LVC vice presidents, board members, teachers, and examiners. As LVC students and future army veterinary surgeons, they followed a curriculum that was modeled on and overlapped with that of human medicine. In their embrace of veterinary medicine, these men were neither the heroes nor the villains described by veterinarian-historians. They were motivated partly by the way that veterinary medicine integrated their preexisting interests in animals, as expressed in the study of equine farriery, agricultural improvement, comparative anatomy and animal experiment. In addition, within the prevailing culture of medico-gentility, the promotion and pursuit of veterinary medicine offered opportunities for them to establish themselves as polite, benevolent, and improving gentlemen. They transmitted these values to the first generation of veterinary students, along with the conviction that veterinary improvement depended upon the participation of medical gentlemen capable of identifying its scientific principles.[Other P-520]

Therefore, in many ways, human and veterinary medicines in Britain at the turn of the nineteenth century were essentially "one medicine." This finding raises a new historical problem. If the foundation of the LVC resulted not—as veterinarian-historians claim—in the establishment of a distinctive veterinary professional domain, but in "one medicine," then how, when, and why did that evolve into the two separate medicines that exist today? This article locates the answer to the problem within early nineteenth-century efforts to reform the knowledge, politics, values, and institutions of (veterinary) medicine. It suggests that veterinarians were the key protagonists, for although medical reformers were engaged in attacking the very people and cultures that had encouraged the late eighteenth-century integration of human and veterinary medicine, they did not abandon their interest in animal bodies and diseases. Indeed, that interest continued to evolve under the impetus of their newly utilitarian, scientifically focused agendas. It meant that when medical men like Thomas Wakley campaigned for LVC reform, they did so in the belief that human medicine and veterinary medicine were "one," whereas veterinarians who embarked on the same objective pursued the ultimate goal of separating veterinary from human medicine and establishing it as an autonomous professional domain.

The ambitions of veterinary reformers like Clark and Youatt grew out a new, collective veterinary identity that developed in the two decades following the Napoleonic Wars. Forged in the context of a competitive marketplace that demanded expertise in the health of various animal species, it grew out of shared experiences of LVC education and veterinary practice, and was fostered by new societies and journals. It involved the construction of a new veterinary epistemology that derived from and contributed to the improvement of veterinary practice. In privileging experience over reason, and species differences over commonalities, this epistemology took farriery and comparative anatomy in directions quite distinct from those pursued by medical men. It also encouraged veterinarians to see themselves as a breed apart: as scientific practitioners who were more educated than farriers, and more practically competent than medical men. These developments meant that although veterinary medicine did not achieve political independence from human medicine until the 1844 award of a Royal Charter, which established its own governing body, by the mid-1830s it had largely assumed its modern identity and characteristics as a field allied to yet distinctly different from human medicine.

Paradoxically, this article reveals that although the ambition to create an autonomous veterinary domain originated among veterinarians, it was human medicine that provided them with the tools for achieving it. In [Other P-521] their efforts to distinguish veterinary from human medicine, veterinary reformers adopted the political goals, rhetoric, and strategies of medical reformers, their epistemological interests, and their preferred modes of education and social organization. Therefore the establishment of veterinary medicine as a domain distinct from medicine resulted, in part, from the very influence that medicine exerted upon it. Moreover, this dynamic persisted. In its subsequent evolution, veterinary medicine followed a very similar—though not uncontested—trajectory to human medicine, involving the pursuit of similar educational standards, legislative privileges, social status, and professional identities.^138^ Its practitioners remained alert to medical encroachments on what they perceived to be their turf, and they continued to assert the distinctive nature of their expertise as derived from experiential knowledge of specific animals, as opposed to the general medical understandings generated through extrapolations across species.^139^ The period 1815 to 1835 was therefore crucial in establishing the somewhat conflicted and paradoxical relationship with human medicine that came to characterize the modern veterinary profession.

These findings have two broad implications. First, they enhance general historical understandings of the professionalization process. In his account of human medicine in this period, Brown critiques the traditional, teleological readings that equate professionalization with the achievement of certain structural and institutional landmarks.^140^ Instead, he emphasizes the cultural process whereby medical men came to imagine themselves as a collective, possessing shared goals, values, and knowledge that were distinct from those of civil society.^141^ This article shows that this process of imagining was not unique to human medicine. In revealing that developments within it inspired vets with overlapping histories to envision themselves likewise as an independent profession, it suggests a possible explanation for how the norms of medical professionalization came to characterize the health professions in general.[Other P-522]

Second, in revealing that the allocation of human and animal health to two discrete professions was not a self-evident development rooted in the biological differences of their patients, but a social historical construct, this article offers insights of relevance to current health initiatives directed at breaking down the boundaries between these domains. Known initially as "One Medicine" and more recently as "One Health," these initiatives are inspired by the shared threats to human and animal health posed by climate change, food insecurity, antimicrobial resistance, and zoonotic diseases. In contrast to the historical "One Medicine" described in this article, its present-day advocates (who are primarily veterinarians) have no intention of merging human and veterinary medicine. Instead they aim to develop collaborative approaches to shared health problems.^142^ The historical record offers some encouragement to their efforts by revealing that animals and their diseases have long interested both doctors and veterinarians.^143^ At the same time, in highlighting the considerable efforts that went into constructing social, educational, epistemological, and political boundaries between human and veterinary medicine, this article reveals why One Health today is proving difficult to implement, for such boundaries cannot be transcended simply through the logic of a shared health problem. Rather, if modern veterinary and medical professionals are to work effectively together, it is necessary for them to understand their shared histories, and the factors that drove the original "one medicine" to divide in two.[Other P-523]

## Footnotes

1
BlaineDelabere, The Outlines of the Veterinary Art; or, The Principles of Medicine: As Applied to the Structure, Functions and Economy of the Horse, the Ox, the Sheep and the Dog (London, 1802), xii.

2 Ibid., viii.

3 Ibid., 107–8.

4 Ibid., 109–11.

5
BlaineDelabere, Canine Pathology; or, A Description of the Diseases of Dogs, 4th ed. (London, 1841), 2–7.

6
MacKayMichael, "The Rise of a Medical Speciality: The Medicalization of Elite Equine Medical Care, 1680–1800" (Ph.D. diss., University of York, 2009). On the relationships between human medicine and early veterinary medicine in France, see HannawayCaroline, "Veterinary Medicine and Rural Health Care in Pre-Revolutionary France," Bull. Hist. Med. 51 (1977): 431–47.334295

7
MacKay, "Rise of a Medical Speciality" (n. 6).

8
BrownMichael, Performing Medicine: Medical Culture and Identity in Provincial England, c.1760–1850 (Manchester: Manchester University Press, 2011).

9
CunninghamAndrew, The Anatomist Anatomis'd: An Experimental Discipline in Enlightenment Europe (Ashgate: Farnham, 2010); GuerriniAnita, Experimenting with Humans and Animals: From Galen to Animal Rights (Baltimore: Johns Hopkins University Press, 2003); GuerriniAnita, "Natural History, Natural Philosophy and Animals 1600–1800," in A Cultural History of Animals in the Age of Enlightenment, ed. SeniorMatthew (Oxford: Berg, 2009), 121–44; RusnockAndrea, "Catching Cowpox: The Early Spread of Smallpox Vaccination, 1798–1810," Bull. Hist. Med. 83 (2009): 17–36.

10
For a summary of recent literature on these topics, see AndrewsJonathan, "History of Medicine: Health, Medicine and Disease in the Eighteenth Century: History of Medicine," J. Eighteenth-Cent. Stud. 34 (2011): 503–15.10.1111/j.1754-0208.2011.00448.xPMC411393125083003

11
CurthLouise Hill, The Care of Brute Beasts: A Social and Cultural Study of Veterinary Medicine in Early Modern England (Leiden: Brill, 2010).

12
WilkinsonLise, Animals and Disease: An Introduction to the History of Comparative Medicine (Cambridge: Cambridge University Press, 1992), 26.

13
Blaine, Outlines of the Veterinary Art (n. 1), viii; MacKay, "Rise of a Medical Speciality" (n. 6).

14
JacynaL. Stephen, "Images of John Hunter in the 19th Century," Hist. Sci. 21 (1983): 85–108; WarnerJohn Harley, "The Idea of Science in English Medicine: The 'Decline' of Science and the Rhetoric of Reform, 1815–45," in British Medicine in an Age of Reform, ed. FrenchRogerWearAndrew (Abingdon: Routledge, 1991), 136–64.

15
CotchinErnest, The Royal Veterinary College (Birmingham: Barracuda, 1990); HallSherwin, "The Struggle for the Charter of the Royal College of Veterinary Surgeons, 1844," Vet. Hist. 8 (1994): 2–21; PughLesley, From Farriery to Veterinary Medicine 1785–95 (Cambridge: Heffer, 1962); PattisonIain, The British Veterinary Profession, 1791–1948 (London: J.A. Allen, 1984). These sources inform the account provided by Wilkinson, Animals and Disease (n. 12), 87–103.

16
Blaine, Outlines of the Veterinary Art, 3rd ed. (London, 1826), v.

17
Blaine, Outlines of the Veterinary Art, 4th ed. (London, 1832), 1–7.

18
Hall, "Struggle" (n. 15).

19
WaddingtonIvan, The Medical Profession in the Industrial Revolution (Dublin: Gill and Macmillan, 1984); LoudonIrvine, Medical Care and the General Practitioner 1750–1850 (Oxford: Clarendon, 1986); WarnerHarley, "Idea of Science" (n. 14); BurneyIan, "Medicine in the Age of Reform," in Rethinking the Age of Reform: Britain, 1780–1850, eds. BurnsArthurInnesJoanna (Cambridge: Cambridge University Press, 2003), 163–82; Brown, Performing Medicine (n. 8).

20 In 1827 the college adopted the epithet "Royal" following the patronage of George IV. However, to avoid confusion, it will be referred to as the LVC throughout this article.

21
DaviesOwen, "Cunning-folk in the Medical Market-Place during the Nineteenth Century," Med. Hist. 43 (1999): 55–73; CurthLouise Hill, "Care of the Brute Beast: Animals and the Seventeenth Century Medical Market-Place," Soc. Hist. Med 15 (2002): 375–92.10.1017/s0025727300064711PMC104411010885133

22
Pugh, From Farriery (n. 15).

23
Mackay, "Rise of a Medical Speciality" (n. 6).

24
HannawayCaroline, "Veterinary Medicine" (n. 6); BolsP. E. J.De porteH. F. M., "The Horse-Catalysed Birth of Modern Veterinary Medicine in 18th-Century France," J. Equine Vet. Sci. 41 (2016): 35–41.

25
The two schools functioned largely independently until the 1844 creation of the Royal College of Veterinary Surgeons. For more on the Edinburgh school, see MacdonaldAlistairWarwickColin, "Early Teaching of the Veterinary Art and Science in Edinburgh," Vet. Hist. 16 (2012): 227–73.

26
MacKay, "Rise of a Medical Speciality" (n. 6).

27
Pugh, From Farriery (n. 15).

28
"Lectures upon the General Knowledge of the Horse," Oracle Bell's New World (London), 12 21, 1789.

29
Pugh, From Farriery (n. 15); Cotchin, Royal Veterinary College (n. 15).

30
"Veterinary College," World, 5 12, 1791.

31
Royal Veterinary College, The First Number of Veterinary Transactions (London, 1801), 45; Pugh, From Farriery (n. 15).

32
BrownMichael, "Medicine, Reform and the 'End' of Charity in Early Nineteenth-Century England," Engl. Hist. Rev. 124 (2009): 1353–88; Brown, Performing Medicine (n. 8), 116–26.

33
Mackay, "Rise of a Medical Speciality" (n. 6).

34
MokyrJoel, The Enlightened Economy: An Economic History of Britain 1700–1850 (London: Yale University Press, 2009), 171–97.

35
Pugh, From Farriery (n. 15); BroadJohn, "Cattle Plague in Eighteenth-Century England," Agric. Hist. Rev. 31 (1983): 104–15. Medical responses to cattle plague in France and its influence on early veterinary education there are described by Hannaway, "Veterinary Medicine" (n. 6) and HannawayCaroline, "The Societe Royale de Medicine and Epidemics in the Ancient Regime," Bull. Hist. Med. 46 (1972): 257–73.

36
LawrenceChristopher, "Alexander Monro Primus and the Edinburgh Manner of Anatomy," Bull. Hist. Med. 62 (1988): 193–214; LawrenceSusan, Charitable Knowledge: Hospital Pupils and Practitioners in 18th Century London (Cambridge: Cambridge University Press, 1996), 318–23; Guerrini, "Natural History" (n. 9); Cunningham, Anatomist Anatomis'd (n. 9), 295–355.3048460

37
Jacyna, "Images" (n. 14); ChaplinSimon, "Nature Dissected or Dissection Naturalised? The Case of John Hunter's Museum," Museum Soc. 6 (2008): 135–51.

38
"Veterinary College," World (London), 4 5, 1791.

39
Brown, Performing Medicine (n. 8), 116–26.

40
"Veterinary College London: For the Reformation and Improvement of Farriery," World, 2 22, 1791, 3.

41
Pugh, From Farriery (n. 15).

42
Blaine, Canine Pathology (n. 5), 4.

43
Moorcroft went on to work for the East India Company in Bengal, and was murdered while exploring the Himalayas. AlderGary, Beyond Bokhara: The Life of William Moorcroft, Asian Explorer and Pioneer Veterinary Surgeon, 1767–1825 (London: Century, 1985).

44
Pugh, From Farriery (n. 15), 80–81.

45 Special Meeting of the Royal Veterinary College Medical Committee, February 17, 1794, RVC Minute Books, Historical Collection, Royal College of Veterinary Surgeons Knowledge, London.

46
"Royal Veterinary College Anniversary Dinner of the Pupils," Farrier and Naturalist 1–2 (1828–29): 196.

47 Royal Veterinary College Council Meeting, January 17, 1795, RVC Minute Books (n. 45).

48
Lawrence, Charitable Knowledge (n. 36).

49
"Veterinary College," Times, 9 15, 1795, 1: Pugh, From Farriery (n. 15).

50
Pugh, From Farriery (n. 15); Cotchin, Royal Veterinary College (n. 15).

51
MacKay, "Rise of a Medical Speciality" (n. 6), 103; Pugh, From Farriery (n. 15), 64.

52
"Medical Theatre, Guy's Hospital," Morning Chronicle, 9 6, 1803, 1; J. F. South, Notes of Lectures on the Veterinary Art Delivered by Edward Coleman esq in the Medical Theatre at Guy's hospital, 1817, MS 1709, Wellcome Library, London.

53
Blaine, Outlines of the Veterinary Art (n. 1), ix; Anon, "Farriery," in Encyclopædia Britannica, vol. 8, 4th ed., ed. MillarJames (Edinburgh, 1810), 418–569, quotation on 419; Mr. Wardrop, "On the Diseases of the Eye of the Horse" (1819), reproduced in Farrier and Naturalist 1–2 (1828–29): 16–17; Anon, "Review of W Percivall, A Series of Elementary Lectures on the Veterinary Art," Med. Chir. Rev. 4 (1823–24): 584.

54 Council meeting (n. 47).

55
"Fifth Session: Seventeenth Parliament of Great Britain," Parliamentary Papers, 4 20, 1795, 447–48; Pugh, From Farriery (n. 15).

56
BoneRobin, "'Veterinary Surgeon,' the First Use of the Title," Vet. Hist. 17 (2015): 430–34.

57
"The Fifth Report of the Commissioners of Military Enquiry," Parliamentary Papers 6 (1808): 127–29; "The Eighth Report of the Commissioners of Military Enquiry," Parliamentary Papers 5 (1809): 147–51.

58
"Veterinary College," Times, 8 13, 1798, 1; Royal Veterinary College, Transactions (n. 31), 46.

59
ColemanEdward, Observations on the Structure, Economy and Diseases of the Foot of the Horse (London, 1798), v–vi.

60
This analysis is based on the following works: Coleman, Observations on the Structure (n. 59); Blaine, Outlines of the Veterinary Art (n. 1); LawrenceRichard, An Enquiry into the Structure and Animal Economy of the Horse, Comprehending the Diseases to Which His Limbs and Feet Are Subject (Birmingham, 1801); RydingWilliam, Veterinary Pathology; or A Treatise on the Cause and Progress of Disease in the Horse, 2nd ed. (London, 1804); BoardmanThomas, A Dictionary of the Veterinary Art (London, 1805); ShippJohn, Cases in Farriery: In Which the Diseases of Horses Are Treated on the Principles of the Veterinary School of Medicine (Leeds, 1806); WhiteJames, A Treatise on Veterinary Medicine, Containing a Compendium of the Veterinary Art, 8th ed. (London, 1807); ClarkBracy, A Series of Original Experiments on the Foot of the Living Horse (London, 1809); Anon, "Farriery" (n. 53).

61
LaneJohn, The Principles of English Farriery Vindicated (London, 1800).

62
Anon, "Farriery" (n. 53), 419.

63 Grooms typically received a tip from the farriers they sent for, and if the horse died, they took possession of the body. However, within the LVC, such payments were outlawed, and dead horses kept for the purpose of student dissection. Royal Veterinary College, *Transactions* (n. 31), vi–viii, xviii.

64
Shipp, Cases in Farriery (n. 60), vi.

65
PooveyMary, Making a Social Body: British Cultural Formation, 1830–1864 (London: University of Chicago Press, 1995).

66
Brown, Performing Medicine (n. 8); Loudon, Medical Care (n. 19); WarnerHarley, "Idea of Science" (n. 14); Burney, "Medicine" (n. 19).

67
BurnsArthurInnesJoanna, "Introduction," in InnesBurns and, Rethinking the Age of Reform (n. 19), 1–70.

68
PladekBrittany, "'A Variety of Tastes': The Lancet in the Early Nineteenth-Century Periodical Press," Bull. Hist. Med. 85 (2011): 560–86; BrownMichael, "'Bats, Rats and Barristers': The Lancet, Libel and the Radical Stylistics of Early Nineteenth-Century English Medicine," Soc. Hist. 39 (2014): 182–209.10.1353/bhm.2011.008522506433

69
Brown, Performing Medicine (n. 8); Loudon, *Medical Care* (n. 19); WarnerHarley, "Idea of Science" (n. 14); Burney, "Medicine" (n. 19).

70 They were Astley Cooper (1768–1841), surgeon to Guy's hospital; John Abernethy (1764–1831), surgeon to St Bartholomew's hospital; Everard Home (1756–1832), brother-in-law to John Hunter and surgeon to St George's hospital; Benjamin Travers (1783–1858), former apprentice to Astley Cooper and a surgeon to St Thomas's hospital; Benjamin Brodie (1783–1862), surgeon to St George's hospital, who was taught by Home and Abernethy; and Joseph Henry Green (1791–1863), nephew to Henry Cline and surgeon to St Thomas's hospital). For their biographies, see the *Dictionary of National Biography*.

71
A Veterinary Surgeon, "The Veterinary College," Lancet 6 (1826): 469–70; Editorial, Lancet 6 (1826): 498–99.

72
"Veterinary College," Morning Chronicle, 7 10, 1829, 1.

73
JonesBruce Vivash, "British Veterinary Periodicals, 1828–1928," Vet. Hist. 17 (2015): 349–71. On Youatt, see ClewlowJohnLockettP., "William Youatt: His Ancestry and Unitarianism," Vet. Hist. 14 (2008): 205–23.

74
Editorial, "The Veterinary College," Lancet 7 (1826): 118–20, quotation on 118.

75
Anon, "The Veterinary College as Originally Constituted; Compared with Its Present Mismanaged and Corrupt State," Farrier and Naturalist 1 (1828–29): 99–105, quotation on 99.

76
WarnerHarley, "Idea of Science" (n. 14).

77 A Veterinary Surgeon, "Veterinary College" (n. 71).

78 Editorial, "Veterinary College" (n. 74), 105.

79
Anon, "Review of E. Coleman, Observations on the Structure, Economy and Diseases of the Foot of the Horse," Lancet 8 (1827): 177–79, quotation on 178.

80
Editorial, "Veterinary College" (n. 74), 103; Editorial, "Veterinary College," Lancet 7 (1826): 185–87.

81 "Veterinary College" (n. 72).

82 Editorial, "Veterinary College" (n. 74).

83
DesmondAdrian, The Politics of Evolution: Morphology, Medicine and Reform in Radical London (London: University of Chicago Press, 1989).

84
PercivallWilliam, A Series of Elementary Lectures on the Veterinary Art (London, 1823), xiv.PMC565217929917562

85
WilkinsonLise, "Glanders: Medicine and Veterinary Medicine in Common Pursuit of a Contagious Disease," Med. Hist. 25 (1981): 363–84; WorboysMichaelPembertonNeil, Mad Dogs and Englishmen: Rabies in Britain, 1830–2000 (Basingstoke: Palgrave Macmillan, 2007).10.1017/s0025727300034876PMC11390697038356

86
JacynaStephen, "Principles of General Physiology: The Comparative Dimension to British Neuro-Science in the 1830s and 40s," Stud. Hist. Biol. 7 (1984): 47–92; Desmond, Politics of Evolution (n. 83).11611372

87
GuerriniAnita, "Animal Experiments and Anti-Vivisection Debates in the 1820s," in Frankenstein's Science: Experimentation and Discovery in Romantic Culture, 1780–1830 ed. KnellwolfChristaGoodallJane (Aldershot: Ashgate, 2008), 71–85.

88
Editorial, "Medical Men and Veterinary Surgeons,"Vet.8 (1835): 580–85.

89
Editorial, *Vet.* 3 (1830): 697–701, 698. The LVC listed 546 qualified vets in its 1831 register, although owing to deaths, this was less than the total number who had qualified since 1791. Royal Veterinary College, Rules and Regulations, with a List of the Subscribers and Names of Veterinary Surgeons (London, 1831).

90 Editorial, "Medical Men" (n. 88).

91 "Veterinary College" (n. 72).

92
GoodwinJoseph, A New System of Shoeing Horses, with an Account of the Different Modes Practiced by Various Nations (London, 1820); Blaine, Outlines of the Veterinary Art, 3rd ed. (n. 16); Anon, 'The Rise and Progress of the Veterinary College of London," Farrier and Naturalist1 (1828–29): 5–12.

93
Blaine, Outlines of the Veterinary Art (n. 1), 91.

94
DawsonRobert, "Veterinary College," Morning Post, 4 10, 1819, 3.

95
Blaine, Outlines of the Veterinary Art, 4th ed. (n. 17), 7–9.

96
Anon, "Royal Veterinary College Anniversary Dinner of the Pupils," Farrier and Naturalist 1 (1828–29): 194–240, quotation on 199.

97
MayerMr., "Professor Coleman and the London Veterinary Medical Society," Vet. 10 (1837): 27–29.

98
LawrenceSusan, "'Desirous of Improvements in Medicine': Pupils and Practitioners in the Medical Societies at Guy's and St. Bartholomew's Hospitals, 1795–1815," Bull. Hist. Med. 59 (1985): 89–104; Brown, "Medicine, Reform" (n. 32), 1380–82.3886052

99
"London Veterinary Society," Lancet 10 (1828): 344–45.

100
"New Veterinary Medical Society," Farrier and Naturalist 1 (1828): 253–56, quotation on 253.

101
ClewlowJohn, "The Mayers of Newcastle-Under-Lyme: Their Contribution to the Emerging Veterinary Profession" (M.Phil. diss., University of Keele, 1995), 42–55.

102
Blaine, Outlines of the Veterinary Art, 3rd ed. (n. 16), 16–17.

103 Ibid., 20.

104
YouattWilliam, "Substance of an Introductory Lecture," Vet. 2 (1829): 6–13; YouattWilliam, "Introductory Lecture on Veterinary Medicine and Surgery," Lancet 17 (1831): 78–82; Editorial, "Introductory Lecture on Veterinary Medicine and Surgery by Mr Youatt," Lancet 17 (1831): 333–37.

105
Editorial, Vet. 7 (1834): 46–49; Lawrence, Charitable Knowledge (n. 36), 162–88.

106
Editorial, "Monstrosities,"Vet.7 (1834): 432; Desmond, Politics of Evolution (n. 83), 81–83, 415.

107
Desmond, Politics of Evolution (n. 83); JacynaStephen, "Immanence or Transcendence: Theories of Life and Organisation in Britain, 1790–1835," Isis 74 (1983): 310–29; JacynaStephen, Philosophic Whigs. Medicine, Science and Citizenship in Edinburgh, 1789–1848 (Abingdon: Routledge, 1994); SparyEmma, "Political, Natural and Bodily Economies," in Cultures of Natural History, ed. JardinNicholasSecordJamesSparyEmma (Cambridge: Cambridge University Press, 1996), 178–96; Burney, "Medicine" (n. 19); Brown, Performing Medicine (n. 8).

108
Blaine, Outlines of the Veterinary Art (n. 1).

109 J Burley, Veterinary Lectures given by Edward Coleman Professor at the Veterinary College, 1809, Historical Collection, Royal College of Veterinary Surgeons Knowledge, London.

110
Coleman, Observations on the Structure (n. 59); ColemanEdward, Observations on the Structure, Economy and Diseases of the Foot of the Horse, vol. 2 (London, 1802); ColemanEdward, Observations on the Formation and Uses of the Natural Frog of the Horse with a Description of a Patent Artificial Frog, to Prevent and Cure Contracted Hoofs (London, 1800).

111 These forges were advertised extensively in the *Times*, e.g., March 21, 1798, 1.

112 Lane, *Principles* (n. 61).

113
PowisRichard, A Concise Dissertation on the Anatomy, Physiology and Most Prominent Diseases of the Foot of the Horse with the Method of Preventing Them or Facilitating Their Cure by the Application of the Levarian or Easy Shoe (London, 1815); Blaine, Outlines of the Veterinary Art, 2nd ed. (London, 1816); ClarkBracy, Stereoplea: The Artificial Defence of the Horse's Foot Considered (London, 1817); Goodwin, SystemNew (n. 92); Anon, "Review of E. Coleman" (n. 79); ClarkBracy, Hippodonomia or the True Structure, Laws, and Economy of the Horse's Foot, and Podophthora or a Ruinous Defect in the Principle of the Common Shoe Detected and Demonstrated by Experiments: with a Proposition for a New Principle of Shoeing Which Abundant Practice Has Since Confirmed (London, 1829).

114 For example, see articles and correspondence in the *Morning Post* (1815), *Sporting Magazine* (1815), *Lancet* (1827–29), and *Farrier and Naturalist* (1828–29), passim.

115
Coleman, Observations on the Structure (n. 59); Coleman, Observations on the Structure, vol. 2 (n. 110); Coleman, Observations on the Formation (n. 110).

116 Clark, *Original Experiments* (n. 60); Clark, *Stereoplea* (n. 113); Clark, *Hippodonomia* (n. 113).

117
Blaine, Outlines of the Veterinary Art (n. 1), 592.

118
Clark, Hippodonomia (n. 113), vi.

119
Desmond, Politics of Evolution (n. 83), 25–100.

120
LockettClewlow and, "William Youatt" (n. 73), 214.

121
YouattWilliam, "Introductory Lecture on Veterinary Medicine and Surgery," Lancet 17 (1831): 78–82.

122
YouattWilliam, The Horse, with a Treatise on Draught (London, 1831); YouattWilliam, Cattle, Their Breeds, Management, and Diseases (London, 1834); YouattWilliam, The Dog (London, 1845).

123 This notion proved more problematic when applied to the relationship between human and animal minds. Jacyna, "Immanence or Transcendence" (n. 107); Jacyna, "Principles of General Physiology" (n. 86).

124
YouattWilliam, On Canine Madness (London, 1830), 17.

125
PercivallWilliam, "Introductory Lecture," Vet. 7 (1834): 7–13.

126 Ibid., 7.

127
Youatt, "Introductory Lecture" (n. 104), 80.

128
Editorial, "Professor Coleman's Introductory Lecture," Vet. 1 (1828): 462.

129 Ibid.

130
LawrenceWilliam, Lectures on Comparative Anatomy: Lecture 2, Introductory to the Course of 1818, 9th ed. (London, 1848), 32. Lawrence drove the *Lancet*'s critique of the London medical elites. His materialistic theory of mind inspired widespread controversy and a charge of blasphemy. Desmond, Politics of Evolution (n. 83), 15.

131
YouattWilliam, "Annual Report of Diseases in Mr Youatt's school," Vet. 7 (1834): 58–60.

132 Editorial, "Introductory Lecture" (n. 128), 461.

133
E.g., Youatt, "Comparative Pathology," Vet. 9 (1836): 23–27, 85–88,

134
Youatt, "Annual Report" (n. 131), 60.

135
Editorial, "Medical Men" (n. 88), 585.

136
Editorial, Vet. 3 (1830): 697–701.

137
Editorial, Vet.7 (1834): 155–61.

138
WoodsAbigailMatthewsStephen, "'Little, If at All, Removed from the Illiterate Farrier or Cow-Leech': The English Veterinary Surgeon, c. 1860–85, and the Campaign for Veterinary Reform," Med. Hist. 54 (2010): 29–54.2004626310.1017/s0025727300004300PMC2793141

139
WorboysMichael, "Germ Theories of Disease and British Veterinary Medicine, 1860–1890," Med. Hist. 35 (1991): 308–27; WaddingtonKeir, The Bovine Scourge: Meat, Tuberculosis and Public Health, 1850–1914 (Woodbridge: Boydell, 2006).10.1017/s0025727300053813PMC10364831886430

140
Carr-SaundersAlexander Morris, The Professions (Oxford: Clarendon, 1933); WeberMax, The Theory of Social and Economic Organization (London: Free Press, 1964); Waddington, Medical Profession (n. 19); PerkinHarold, The Rise of Professional Society (London: Routledge, 1989).

141
Brown, "Medicine, Reform" (n. 32).

142
CassidyAngela, "One Medicine? Advocating (Inter)disciplinary at the Interfaces of Animal Health, Human Health, and the Environment," in Investigating Interdisciplinary Collaboration: Theory and Practice across Disciplines, ed. Scott Frickel, Mathieu Albert, and Barbara Prainsack (New Brunswick, N.J.: Rutgers University Press, 2016, 213–35).27854405

143
BresalierMichaelCassidyAngelaWoodsAbigail, "One Health in History," in One Health: The Theory and Practice of Integrated Health Approaches, ed. ZinsstagJacobSchellingEstherWaltner-ToewsDavidWhittakerMaxineTannerMarcel (Wallingford: CABI, 2015), 1–15.

